# The Current State of the Art in PARP Inhibitor-Based Delivery Nanosystems

**DOI:** 10.3390/pharmaceutics14081647

**Published:** 2022-08-08

**Authors:** Lisha Cai, Xiaoling Xu, Wei Chen

**Affiliations:** 1 Cancer Institute of Integrated Traditional Chinese and Western Medicine, Key Laboratory of Cancer Prevention and Therapy Combining Traditional Chinese and Western Medicine of Zhejiang Province, Zhejiang Academy of Traditional Chinese Medicine, Tongde Hospital of Zhejiang Province, Hangzhou 310012, China; 2 Shulan International Medical College, Zhejiang Shuren University, Hangzhou 310015, China

**Keywords:** PARP inhibitor, nanosystem, drug delivery, cancer therapy

## Abstract

Poly (adenosine diphosphate [ADP]–ribose) polymerases inhibitors (PARPi), the first clinically approved drug that exhibits synthetic lethality, are moving to the forefront of cancer treatments. Currently, the oral bioavailability of PARPi is quite low; thus, it is a major challenge to effectively and safely deliver PARPi during clinical cancer therapy. Nanotechnology has greatly advanced the development of drug delivery. Based on the basic characteristics and various forms of nanoparticles, drug delivery systems can prolong the time that drugs circulate, realize the controlled release of drugs, provide drugs with an active targeting ability, and spatiotemporally present combination treatment. Furthermore, nanosystems may not only enhance drug efficiency but also reduce adverse side effects. This review focuses on strategies involving nanoparticle-based delivery for PARPi, including single administration and codelivery with other agents. We believe that nanosystems have great potential in advancing PARPi efficacy for cancer therapy.

## 1. Introduction

DNA damage includes DNA single-strand breaks (SSBs) and double-strand breaks (DSBs). Poly (adenosine diphosphate [ADP]–ribose) polymerases (PARP) are a large family of multifunctional enzymes that are closely associated with DNA damage repair (DDR) [1,2]. SSBs are mostly repaired via a base excision mechanism, in which PARP1/2 both exert critical impacts. The repair of DSBs largely relies on a homologous recombination (HR) mechanism, in which the tumor-suppressor proteins BRCA1 and BRCA2 play crucial roles [1,3,4]. In addition, PARP1 plays a vital role in the repair of DSBs and damage to the replication fork [5]. Therefore, in the presence of PARP inhibitors (PARPi), the repair of SSBs fails, thereby resulting in an accumulation of SSBs and further replication fork of DSBs [3,6]. Furthermore, it has been stated that PARPi exhibit the ability to trap PARP enzymes on DNA and prevent the process of DDR, which eventually leads to cell death [7,8]. Hence, due to this cytotoxic activity, PARPi is a potent candidate for cancer therapy.

To date, four PARPi have been approved for the clinical treatment of cancers with germline BRCA mutations by the US Food and Drug Administration (FDA), and these PARPi include olaparib, talazoparib, rucaparib, and niraparib [9,10]. During treatment, cancer cells with deficient HR DNA repair are found to exhibit striking sensitivities to PARP inhibition, and the activity of PARPi in patients with BRCA mutant cancers is clearly observed [2,3,11,12]. Hence, PARPi have been demonstrated to be an effective therapeutic strategy against cancers with deficient HR DNA repair. The performance of synthetic lethality in cancer therapy has rapidly accelerated its clinical development and extended its application scope [10,13]. Additionally, PARPi has also exhibited therapeutic efficiency on wild-type tumors, and various clinical trials involving PARPi for tumors without HR deficiency are being conducted [1,11,14]. However, the clinical efficacy of the present PARPi is far from ideal, mainly for the following reasons: 1. PARPi are mainly orally administered [15,16]. All orally administered drugs must undergo first-pass metabolism, which greatly reduces drug bioavailability [17,18,19]. For instance, in the form of tablets and capsules, olaparib exhibits a bioavailability of only 25~50% after first-pass metabolism occurs [20,21,22]. 2. The tumor tendency and selectivity of PARPi are weak, and thus the drug accumulation in tumor tissues is relatively low, resulting in poor efficacy [23]. 3. Given the complexity of the tumor microenvironment, the efficacy of PARPi monotherapy is inadequate [24]. Thus, there is a compelling need to determine how to safely and effectively deliver drugs to tumor tissues.

The introduction of nanotechnology has largely overcome the above limitations. Nanotechnology commonly refers to structures that are up to several hundred nanometers in size [25]. In recent decades, a number of nanoformulations, as drug delivery systems (DDSs), have been reported for the treatment of cancers, such as liposomes, polymeric micelles, hydrogels, nanoemulsions, nanosuspensions, and nanoparticles (NPs) [26]. NPs include inorganic, polymeric, and solid lipid nanoparticles (SLNs), like iron oxide (IO), metal–organic frameworks (MOF), poly (lactic-co-glycolic acid) (PLGA), and poly (ε-caprolactone)-poly (ethylene glycol)-poly (e-caprolactone) (PCEC) NPs. In cancer therapy, the advantages of nanosized DDSs have been robustly explored and proven [27,28]. In general, DDSs have been shown to carry drugs to the target sites through enhanced permeability and retention (EPR) effect/active targeting to release the drug precisely, so as to achieve better therapeutic effects and reduce drug toxicity and side effects. Meanwhile, DDSs could also control the release behavior of drugs to maintain the stability of the blood drug concentration in the organism, and further improve the pharmacokinetic parameters and bioavailability [27,28]. Specifically, first of all, DDSs could improve the bioavailability of drugs. NPs could provide protection for drugs in blood circulation and prolong their circulation time [29,30]. In a recent study, compared to free zingerone, the oral bioavailability of zingerone was found to be enhanced by 5.1 times after loading in the nanomicelle DDS [31]. Second, DDSs could also improve the ability of drugs to target tumor sites by modifying ligands, antibodies, oligonucleotides, and even cell membranes [32,33]. Feng et al. extracted HeLa cell membranes to encapsulate nanospheres loaded with Doxorubicin (Dox). When Dox was modified by cell membranes, there was a remarkable enhancement in cellular uptake by HeLa cells because of the homologous targeting of cell membranes [34]. Lastly, multipath treatment and combined therapy could be achieved via DDSs [35]. Various nanoformulations allow drugs to be delivered via multiple routes, including oral administration, injection, and transdermal administration, which could change the current requirements for PARPi of a single administration. In addition, the codelivery of different kinds of drugs can be easily accomplished through DDSs to achieve synergistic effects via combination therapy [36,37].

**Figure 1:**
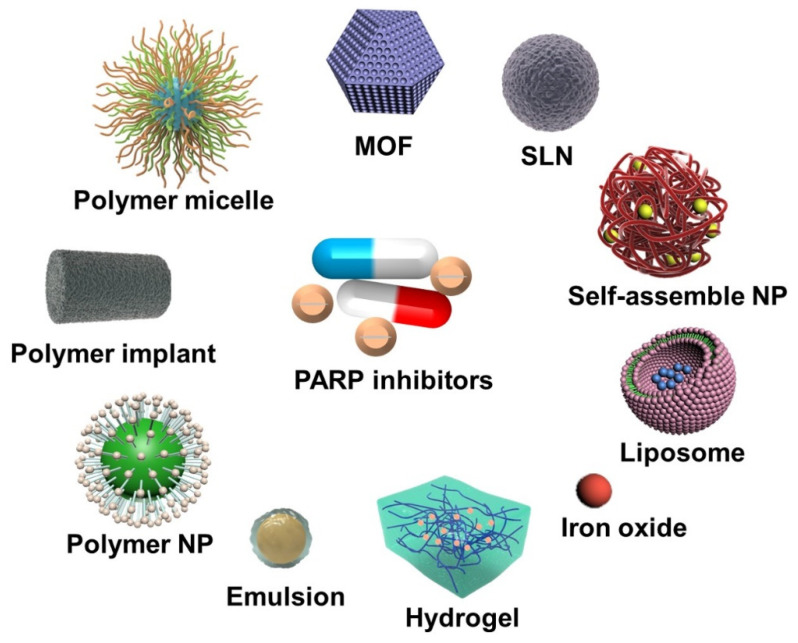
Different types of nanosystems for the delivery of PARP inhibitors in this review. (PARP: poly (adenosine diphosphate [ADP]–ribose) polymerases; MOF: metal-organic framework; SLN: solid lipid nanoparticle; NP: nanoparticle).

In this direction, a multitude of PARPi delivery nanosystems are currently under investigation and are expected to exhibit excellent performance in future clinical therapy [38,39]. This review describes strategies for delivering PARPi with different kinds of nanosystems (Figure 1), such as PARPi monotherapy, codelivery with chemotherapeutic drugs, and combined therapy with radiotherapy (RT)/photodynamic therapy (PDT). The great results of specially designed delivery nanosystems are also highlighted.

## 2. Nanoparticle-Based PARP Inhibitor Delivery Systems

### 2.1. PARPi Monotherapy

In the therapeutic currently used, PARPi are generally administered orally with a high frequency, which leads to adverse side effects including anemia and fatigue [23,40]. For instance, olaparib capsules are orally administered for chemotherapy at the maximum tolerated dose (400 mg b.i.d.) because the pharmacokinetics of olaparib are far from ideal, and only low percentages of the drugs reach the tumor sites, which forces patients to swallow up to 16 capsules per day to maintain effective drug concentrations [41,42]. Therefore, up to 80% of patients experience one or more adverse events [42]. Herein, nanotechnology is employed and has led to a huge shift in cancer therapy. Compared to daily oral dosing, controlled PARPi delivery nanosystems have the potential to reduce systemic toxicity and improve patient outcomes at clinically equivalent doses or below, thus offering the possibility of decreasing the dosing frequency [23]. Hence, nanoformulations of PARPi are actively being explored, and here we demonstrate that the developed nanoparticle-based delivery of PARPi could address the challenges faced by oral administration. 

#### 2.1.1. Olaparib-Based Nanosystems

Olaparib, as a first-in-class and orally active small molecular inhibitor, was the first PARPi that demonstrated superior efficacy and tolerability compared to that of standard chemotherapy in BRCA-mutated breast cancer [11,43]. In this regard, olaparib was also the first PARPi approved by the FDA and the European Medicines Agency (EMA) for the treatment of solid tumors [43]. By taking advantages of nanoformulations as drug carriers, the efficacy and potential of olaparib in conventional therapy have significantly expanded.

Akshay et al. prepared different olaparib nanoformulations of lipospheres (OLA-LP) via the melt dispersion method, and nanosuspensions via the solvent evaporation (OLA-NSP_SE_) and wet milling (OLA-NSP_WM_) techniques [15]. The results of pharmacokinetic parameters showed that the area under the curve (AUC) of the nanoformulations was increased by 1.5 (OLA-LP), 1.9 (OLA-NSP_SE_) and 1.4-fold (OLA-NSP_WM_), respectively, in comparison with olaparib suspension. Moreover, improvement in the index of oral bioavailability was also observed in all nanoformulations, displaying the promise of nanoformulations to decrease the dosing frequency and dose limitation in clinical application.

In one interesting study, injectable nano-olaparib was fabricated via a thin film hydration method to overcome the issue of bioavailability. The size of the prepared nano-olaparib was assessed at 72.8 ± 5.8 nm, with a polydispersity index of 0.218, and zeta potential estimated at −30.5 ± 9.0 mV. When ~90% of oral olaparib was released within 4 days, the nano-olaparib exhibited a first-order drug release profile of 100% over 8 days. When the nano-olaparib was delivered intraperitoneally and daily at the same dose as that for orally administered olaparib, the results confirmed that nano-olaparib offered a better efficacy than that of oral olaparib. In biodistribution profiles, nano-olaparib started accumulating in tissues within 1 h and persisted for ≥72 h after a single dose administration. Moreover, a variable response to oral olaparib was observed, but the response variability decreased noticeably in nano-olaparib that was delivered intraperitoneally. However, reduced tumor growth and systemic toxicity were both observed after the daily administration of nano-olaparib. The authors attributed the remaining toxicity to the nanoformulation since it was also exhibited by the empty NPs that were administered daily. The researchers further proved that performing surface modifications, such as modifying a bioadhesive surface or increasing target moieties, was a promising strategy to enhance the effectiveness of the nano-olaparib [44].

Although the passive targeting helped the above NPs to prolong the drug circulation time, the recognition capability to tumor sites was still largely limited. Therefore, researchers further modified various ligands, antibodies, and oligonucleotides on the surface of NPs to improve the active targeting ability for selected tumor cells, and to increase the accumulation in tumor sites [45]. As a natural tumor target, H-Ferritin is mediated by transferrin receptor-1 (TfR1) and is expected to target the cell nucleus. Herein, H-Ferritin nanoformulated olaparib (HOla) was designed to exert effects on triple-negative breast cancer (TNBC) cell lines in which TfR-1 was overexpressed. HOla was prepared by two different procedures: the disassembly/reassembly method and drug complexation with Cu(II). And HOla obtained by Cu(II)-mediated loading strategy showed cave sphere structures with a size of 14.3 ± 4.2 nm and zeta potential of −20.5 ± 1.2 mV, resulting in 7-fold increase in olaparib loading yields compared to that of the disassembly/reassembly procedure. As the results showed, HOla was rapidly internalized into TNBC cells after the accurate identification of H-Ferritin. More olaparib thus entered the nuclear compartment of cells with the help of H-Ferritin in NPs, resulting in the enhancement of PARP-1 cleavage and DSBs. For both BRCA-mutated and nonmutated TNBC cells, HOla exhibited a strong cytotoxic effect, with 1000-fold higher anticancer activity than that of the free olaparib. Hence, in olaparib monotherapy, cytotoxic efficacy could be significantly enhanced by promoting targeted nuclear delivery via nanoformulation and H-Ferritin modification [46].

**Figure 2:**
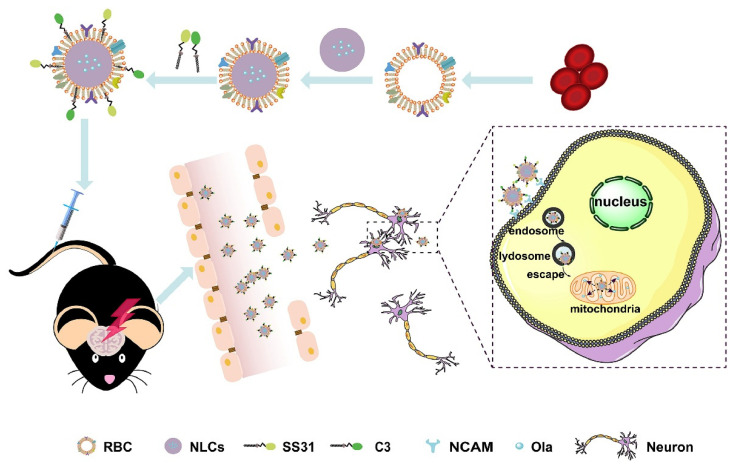
Schematic illustration of erythrocyte membrane-coated nanostructured lipid carriers (RBCNLCs) co-modified with C3 and SS31 peptide for brain neuronal mitochondria targeting to reduce the negative effects of mitochondrial PARP activation. (RBC: red blood cell; NLCs: nanostructured lipid carriers; NCAM: neural cell adhesion molecule; Ola: olaparib). Reprinted with permission from Ref. [50]. Copyright 2022 Elsevier, under the license number 5358651488895.

Since nanomaterials themselves may cause toxicity, as mentioned above, biomimetic nanosystems offer a safer approach, and have recently become the focus of attention and extensive research [47,48]. Red blood cell membranes represent a potential cell-mediated drug delivery platform due to their inherent biocompatibility, biodegradability, and long circulating half-life [48,49]. Accordingly, a red blood cell membrane was employed to coat nanostructured lipid carriers of olaparib. After C3 and SS31 peptides were further dual-modified, the red blood cell membrane-coated nanostructure lipid carriers with C3 and SS31 (termed C3/SS31-RBCNLCs-Ola nanosystem) was prepared in a final size of 126.23 ± 0.94 nm and a zeta potential of −9.66 ± 0.43 mV. With the prolonged circulation time of the red blood cell membrane and the brain neuronal mitochondria target of the C3 peptide, olaparib was largely delivered into brain mitochondria and was maintained at a high concentration by C3/SS31-RBCNLCs-Ola, which effectively prevented the neuronal cell death caused by excessive activation of injury-induced mitochondrial PARP (Figure 2). Therefore, olaparib displayed a greater neuroprotective effect when its biomimetic nanostructure and functional target ligands were utilized, indicating the huge potential of nanodrugs in future clinics [50].

#### 2.1.2. Talazoparib-Based Nanosystems

It has been widely acknowledged that compared to olaparib, the next-generation PARPi talazoparib (BMN-673, recommended 1 mg orally once daily) is a more potent drug [51]. Talazoparib is known for its ability to exhibit a high PARP inhibition with potency at much lower concentrations than that of olaparib [52,53]. Baldwin et al. also demonstrated that a 20-fold decrease in colony formation of PC-3 cells occurred between talazoparib and olaparib at the lowest treatment dose (0.5 μM) [54]. However, the hydrophobic nature and oral delivery route of talazoparib still decreased its bioavailability and led to off-target toxicity in clinical practice [52,53].

Polymeric NPs are made of biocompatible and biodegradable polymers [55]. A novel delivery system for talazoparib was designed by Jodi et al. Talazoparib (25 μg/mm) was loaded in PLGA implants (800 nm diameter) by extrusion followed by curing. Through tuning the PLGA formulation, the localized, slow, and sustained release of talazoparib, which lasted ~28 days, was achieved both in vitro and in vivo. In addition, BRCA1-deficient W780 and W0069 breast cancer cells both exhibited significant sensitivities to growth inhibition by talazoparib implants in vitro. After the in vivo talazoparib implant treatment, which was performed via a one-time intratumoral injection, tumor growth was remarkably slowed (14-fold compared to that of the control), the survival time was prolonged 2-fold, less weight loss was observed, and no acute or chronic toxicity was observed compared to that of the controls. Additionally, the immunohistochemistry results showed that PARP inhibition led to more DSBs and less tumor cell proliferation in talazoparib implant-treated mice [56]. Hence, talazoparib implants were thought to be an easy and potential way to provide superior drug effectiveness, with higher efficacy and lower systemic toxicity.

Lipid carriers refer to nanocarriers composed of lipids or phospholipids, including liposomes, SLNs, nanostructured lipid carriers (NLCs), and nanoemulsions [57,58]. Liposomes, as one of the prominent nanocarriers, have resulted in tremendous advancements for drugs in clinical therapy [59]. Liposomes have structures that are similar to those of biological membranes, which makes it feasible to deliver drugs via permeation [60]. Herein, talazoparib was incorporated into the bilayer of nano liposomes, and the system was termed NanoTLZ. NanoTLZ was stable in size (74.5 ± 11.0 nm) with zeta potential (15.3 ± 1.6 mV) at 4 °C for 2 months. Compared to the saline control, empty NPs, and free talazoparib groups (oral and i.v.), BRCA-deficient mice presented statistically prolonged progression-free survival and overall survival with NanoTLZ treatment. The nanoformulation not only significantly enhanced the efficacy of talazoparib but also reduced extra organ toxicity. In addition, compared to all other experimental groups, NanoTLZ was found to be better tolerated, as less weight loss and alopecia were observed. For the pharmacokinetics of NanoTLZ, the plasma data fit a two-compartment model with a terminal half-life of 37.5 h, and the PAR level was significantly decreased 30 min after injection, remaining at lower levels than control tumors up to 72 h post-injection. Moreover, NanoTLZ preferentially altered the expression of over 140 genes, induced DNA damage and cell cycle arrest, inhibited the proliferation of tumor cells, and further modulated immune cell populations, which finally achieved complete tumor regression [61].

Another nanoemulsion formulation of talazoparib (TZNE) was designed and optimized to 151.4 ± 0.7 nm, with a zeta potential of −33.30 ± 1.22 mV. In drug-resistant cancer cell lines, high intracellular uptake of TZNE with strong fluorescence intensity was visualized. In addition, significant cytotoxicity and induced apoptosis were observed, which was concentration-dependent and far superior to that of the free drug [62].

However, PARPi also faced resistance like other chemotherapy drugs. To date, PARPi resistance has been common and developed through multiple mechanisms, such as HR restoration, reversion mutations, and protection of the DNA replication fork [16,63]. In reported studies, dysregulation of miRNA expression has been shown to be associated with chemoresistance [63]. Hence, talazoparib was designed to be loaded into SLNs with miRNAs (miR-107, miR-193b and miR-1255b). The prepared nanosystem had an ~85.0% entrapment efficiency of talazoparib and a sustained drug release for 12 days. The in vitro study demonstrated that SLN formulation remarkably improved anticancer efficacy in TNBC cells. Moreover, collected data suggested that miRNAs regulated HR restoration and increased RAD51 and BRCA1 expression in resistance cells; thus, the SLN nanosystem overcame the acquired resistance by altering the expression levels of genes and miRNAs in the HR mechanism [64].

#### 2.1.3. Others

Benzofuran–pyrazole compound (BZP), as a PARP1 enzyme inhibitor, was demonstrated to result in a more favorable impact when in the nanosized particles form (termed BZP-NPs). BZP was synthesized by Manal et al., and nanosized BZP-NPs of different sizes (3.8–5.7 nm) were also prepared via the nanoprecipitation method. As expected, BZP-NPs exhibited a greater induction of pre-G1 apoptosis and cell cycle arrest at G2/M phase than that of BZP, resulting in the enhancement of cytotoxicity against MCF-7 and MDA-MB-231 cells (human breast cancer cells) [65]. The preference of NPs with passive targeting as drug carriers was thus clearly displayed.

An active targeting nanosystem is also presented here. Plectin is highly expressed and localized on the cell surface of advanced ovarian cancer, and the plectin-targeting peptide (PTP) has been demonstrated to specifically target cell surface plectin. Herein, PTP-conjugated nanoliposomes with the PARPi AZ7379 encapsulated inside were prepared, with a size ranging between 110–120 nm and a zeta potential of 31 ± 1.6 mV. In the treatment of mice bearing subcutaneous and intraperitoneal advanced ovarian cancer tumors (SKOV3 and OVCAR8), AZ7379 incorporated in PTP liposomes profoundly enhanced PARP inhibition, leading to deceleration of tumor growth and a 3-fold and 1.7-fold decrease in tumor volumes [66]. And compared to the no peptide liposomes, a 1.3- and 1.9-fold greater AUC of PTP liposomes was observed in SKOV3 and OVCAR8 tumors, respectively, via a two-compartment model fit. Therefore, actively-targeted drug delivery assisted by nanomaterials was proven to magnify the efficacy of drugs.

### 2.2. Combined Therapy

Since a complicated network of signaling pathways is involved in cancer therapy, the combination of various therapies is essential [67]. The rationale for combination therapy works via different mechanisms, thereby enhancing tumor cell killing over single treatments [68,69]. There are many combined therapies of small molecular inhibitors with other agents, which have been confirmed to exhibit enhanced efficacy in different types of cancer [68]. At present, the combination of PARPi with chemotherapy and radiotherapy has been extensively explored for synergistic effects [70,71]. However, the additional toxicity and drug multi-resistance in response to combined drugs are also rapidly increasing, seriously influencing the lives of patients [72]. Nanotechnology is obviously capable of eliminating these obstacles. With the help of nanoformulations, the therapeutic efficacy of drugs could be further maximized by synchronizing pharmacokinetics and biodistribution, while the multidrug resistance and systemic toxicity could be simultaneously reduced [23,27].

#### 2.2.1. Chemotherapeutic Drugs

Recently, a number of studies on combinations of PARPi and chemotherapy drugs have been reported [38]. Chemotherapy drugs are mostly able to affect protein synthesis by interacting with signaling pathways, while PARPi potentially inhibit DNA repair, which synergistically acts on cell behaviors and increases therapeutic efficacy [9]. Additionally, the results demonstrated that blocking DNA repair by PARPi had the potential to sensitize tumor cells to antitumor drugs, to overcome the resistance of tumor cells and further improve the effectiveness of combined treatments. For instance, a treatment strategy involving the PARPi olaparib led to a high response rate in patients with prostate cancer, in which the patients no longer responded to single taxane drugs [41]. The results of clinical trials also demonstrated that PARPi tend to sensitize tumor cells to antitumor drugs to overcome platinum-based drug resistance [72]. Unfortunately, grade III or IV adverse events, including anemia and fatigue, were also reported by 20% of patients during the combined therapy [41]. Therefore, based on the codelivery of PARPi and antitumor drugs, nanoformulations and functional modification are equally essential in combined therapy. Some designed combination nanosystems are presented here for clarity.

##### Hybrid Nanosystems

For olaparib, novel localized drug delivery systems were constructed in the form of hydrogels, liposomes and PLGA NPs with etoposide [73], carboplatin [74], and paclitaxel (PTX) [75]. Based on the combination of antitumor drugs and olaparib, all these hybrid nanosystems displayed efficacious performances in the treatment of solid tumors [73,74,75].

For example, etoposide and olaparib were co-delivered by NPs based on bioadhesive hydrogels, which were further coated with polylactic acid-polyethylene glycol to achieve better stability. A sustained and controlled release of drugs for over 120 h was achieved in vitro. When the drugs were administered directly into surgical resection sites of the brain parenchyma with Pluronic F127 spraying, bioadhesion to the brain and gelling at physiological brain calcium concentrations were observed. The fluorescent images with the NPs presented in the tissue surrounding the resection cavity provided convincing evidence that drugs accumulate in the brains of large mammals with no neurotoxicity. All collected data indicated that the developed nanosystem augmented therapeutic efficiency in the treatment of malignant brain tumors [73].

##### Self-Assembled NPs

Although nanomaterials significantly improved the efficacy of PARPi, the introduction of carrier polymer materials in the preparation process may cause long-term toxicity to some degree in vivo [76,77]. Carrier-free NPs were therefore developed, which could self-assemble into nanoparticles by simply adjusting parameters, such as the ratio between drugs [78,79]. The self-assembled NPs not only entered and remained in tumor tissues through the EPR effect, which was mediated by their nanosize, but were also endowed with higher biocompatibility without the introduction of polymer materials [79,80].

**Figure 3:**
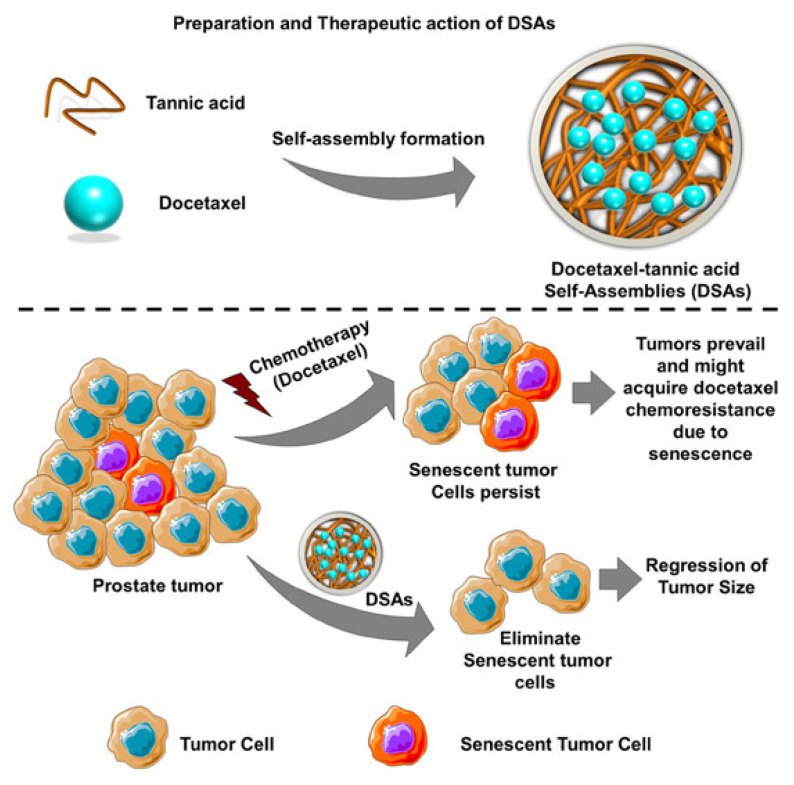
Schematic representation of DSA preparation and the antisenescence mechanism of DSAs in prostate cancer. (DSAs: Docetaxel−tannic acid self-assemblies). Reprinted with permission from Ref. [81]. Copyright 2019 American Chemical Society.

Tannic acid (TA), as a PARP-1 inhibitor, has been reported to reduce PARP-1-mediated cell death. Docetaxel−tannic acid self-assemblies (DSAs) based on nanoformulations were developed to target and remove senescent cells from prostate tumors (Figure 3). The DSAs were prepared by the conventional solvent evaporation and extrusion method and exhibited 95.42 μg of docetaxel for 1 mg of TA-containing formulation. The average particle size and surface charge of optimum DSAs in water were 87.78 ± 1.91 nm and −15.1 ± 0.4, respectively. Compared with free docetaxel treatments in vitro, the DSAs significantly improved cellular internalization, suppressed cell senescence by deregulating TGFβR1/FOXO1/p21 signaling, and induced cell death via activation of apoptotic signaling. The DSAs were found to effectively target tumors 72 h post-injection in all mice. Moreover, the profound antisenescence and anticancer activity of DSAs was observed and further evidenced by the superior tumor-target action and the regression of tumor growth in the PC-3 xenograft mouse model. Hence, the DSA nanoformulation facilitated effective delivery and promoted the efficacy of docetaxel in prostate cancer cells [81].

Similarly, stable TA-PTX NPs (TAP NPs) were alternatively prepared via self-assembly for chemo-sensitization. TAP NPs displayed a ~96% encapsulation efficiency of PTX and offered a higher drug concentration at the target via intracellular drug uptake, resulting in a better therapeutic efficacy and fewer side effects compared to those of free PTX [82].

##### Active Targeted NPs

Although the above two types of NPs could enter tumor tissues, the drugs delivered via the EPR effect alone do not result in a sufficient efficacy. Therefore, the modification of active targeting is very much needed to further improve the drug delivery efficiency.

**Figure 4:**
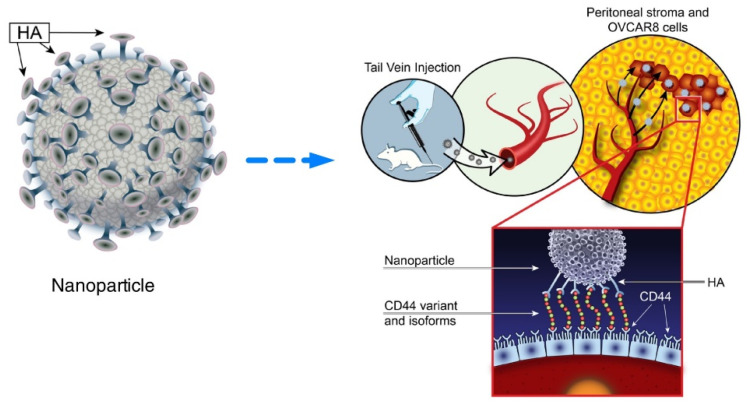
Schematic illustration of the design of the polymeric liposomal nanoparticle assembly with loaded therapeutic cargo and the treatment mechanism. (HA: hyaluronic acid). Reprinted from Ref. [84] under the terms of the Creative Commons CC BY license.

CD44 is a transmembrane adhesion glycoprotein that is highly overexpressed on most ovarian cancer cells; thus, its specific ligand hyaluronic acid (HA) is frequently employed as an active target for the CD44 receptor [83]. Here, an active targeted nanosystem was developed via layer-by-layer liposomal NPs (LbL NPs), with both PARPi and cisplatin loaded within the core and the HA layer equipped outside (Figure 4). The NPs were prepared with a mean diameter of 90 ± 12 nm and a final net surface charge of −31 ± 6 mV. The difference in therapeutic effects between nonincorporated drugs and those combined with LbL NPs was investigated in vivo. LbL NPs extended the survival and blood circulation half-life and provided an ability to actively select targets for serous ovarian cancer cells via CD44. Since the active targeting of NPs, drugs were found to be colocalized within tumor sites and to be time-dependently decreased in organ accumulation, leading to a reduction in hematological and systemic toxicity. In the dual-drug release profiles of LbL NPs, PARPi was released first as expected and then by cisplatin. In this way, the first released PARPi downregulated PARP protein activity and induced SSBs accumulation to sensitize tumor cells, which was beneficial for subsequently released cisplatin to induce more DSBs and cell death. This view was consistent with results that LbL NPs remarkably regressed tumor growth, increased tumor remediation, and decreased tumor metastasis in orthotopic tumor xenografts [84]. The NPs amplified the effect of both drugs and addressed many challenges involved in free drug delivery.

An epidermal growth factor receptor (EGFR) target was also employed for the codelivery of gemcitabine and olaparib. The prepared NPs (GENP) exhibited a strong selectivity to cells with high expression levels of EGFR. In capan-1 tumor xenograft mice, pharmacokinetic results demonstrated that GENP could prolong the circulation time of gemcitabine and olaparib, facilitate coordinated drug release, and reduce the distribution of drugs in major organs. In addition, olaparib of GENP was found to enhance the cytotoxic effect of gemcitabine so that the two exhibited strong and synergistic inhibition of tumor cell growth [85].

#### 2.2.2. Radiotherapy

Up to now, RT has been commonly utilized in the clinical treatment of cancers [86]. Additionally, chemoradiation has been stated to be an effective combination candidate as a therapeutic approach to magnify individual powers and expand therapeutic windows [87]. Hence, merging PARPi with chemoradiation is also a promising strategy for increasing DNA damage and promoting tumor cell death [88]. A few of these combinations have been conducted and cited in this review.

##### Olaparib-Based Nanosystems

Injectable olaparib NPs (Ola-NPs) were designed as potent radiosensitizers to enhance the sensitization and therapeutic effect of RT. The Ola-NPs had a mean size of 31.96 ± 1.54 nm, with a polydispersity index of 0.126 ± 0.014. As the results showed, Ola-NPs displayed an outstanding radiosensitization effect, with a sensitization enhancement ratio of 3.81, which was much higher than the 1.66 of free olaparib. Additionally, the combination of Ola-NPs and RT powerfully inhibited tumor growth and prolonged the median survival time (69.5 ± 11.8 days vs. 31.8 ± 6.7 days) in mice with human non-small-cell lung cancer xenograft tumor models. In addition, Ola-NPs increased cell numbers in the G2/M phase and decreased the angiogenesis of tumor tissues without causing additional cytotoxicity to normal tissues. The mechanism of radiosensitization and thereby improved antitumor efficacy was investigated and attributed to the inhibition of DSBs repair and promotion of cell apoptosis by Ola-NPs [89].

In another approach, olaparib was nanoformulated into a lipid-based injectable nano-olaparib. In the PTEN/TP53-deficient mouse tumor model, there was 19-fold higher NP accumulation in nano-olaparib-treated tumors than in untreated and radiation-only controls. Under treatment with nano-olaparib combined with RT, the survival time of mice was 3-fold longer than that of RT-only controls, and half of the mice tolerated the treatment well and completed the whole therapy [42].

Based on above passive target nanosystems, actively targeted NPs were further developed to achieve a higher targeting ability. The folate (FA) receptor, as a coupling protein, is overexpressed on the surface of many tumor cells. Based on this characteristic, imaging agents and therapeutic drugs can be coupled with FA to actively target tumor cells, and this method has been widely used in tumor imaging diagnosis and cancer treatment [90]. Herein, FA-conjugated PCEC copolymer was employed to develop a sustained release system of olaparib, which ultimately formed active targeting olaparib NPs (ATOs, 240.62 ± 8.79 nm, −10.5 ± 1.09 mV). The in vitro results suggested that ATO powerfully inhibited cancer cell growth by delivering more olaparib into HeLa cells via FA-mediated endocytosis. The combined antitumor effect of ATO and RT was also evaluated through cervical cancer xenograft models. The data demonstrated that ATO had a strong ability to target HeLa cells via FA and thus was taken up by cells, leading to a high concentration of olaparib in the cells and outstanding efficacy. Furthermore, ATO+RT prominently inhibited cell growth with the increased induction of DSBs and cell apoptosis and prolonged the survival time while resulting in no extra toxicity to organs [91].

##### Talazoparib-Based Nanosystems

**Figure 5:**
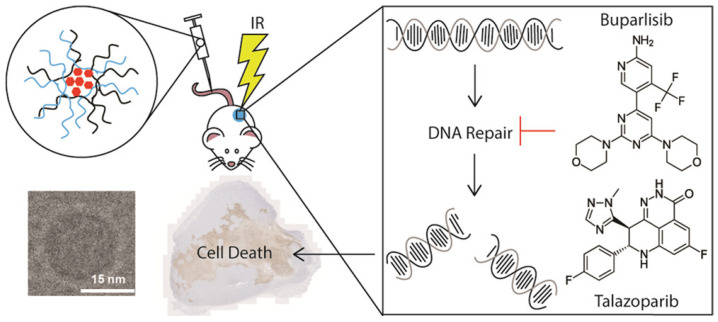
Schematic illustration of the design of MPM with loaded talazoparib and buparlisib, and the combined administration with radiation for enhanced DNA damage. (MPM: mixed poloxamer micelle; IR: irradiation). Reprinted with permission from Ref. [92]. Copyright 2019 American Chemical Society.

Talazoparib and buparlisib were highly incorporated into a novel mixed poloxamer micelle (MPM) nanosystem (Figure 5), which comprised monodisperse sub-20 nm particles with excellent stability and versatility. The drug release profile in vitro was found to be pH dependent, and this system tended to release more drugs in an acidic tumor environment. When encapsulated in MPM, talazoparib and buparlisib exhibited synergistic action in cytotoxicity to cells and improved the radiosensitivity. In a 4T1 murine breast cancer model, collected data demonstrated that increased DNA damage and more cell apoptosis were caused by this combined therapy, which thus delayed tumor growth and prolonged overall survival. Additionally, there was no reduction in particle accumulation upon radiation, and no acute or chronic toxicity were observed during the whole course, which indicates that the nanosystem is very helpful [92].

In another study, talazoparib and buparlisib were loaded into nanoscale MOF. The NPs + RT exhibited sustained induction of DNA damage in vitro, leading to a synergistic enhancement of cytotoxicity and radiosensitivity. Moreover, the combined therapy resulted in the highest induction of cell apoptosis and inhibition of cell proliferation in the rodent model in vivo [93].

#### 2.2.3. Photodynamic Therapy

PDT has been stated to be an alternative cancer treatment with fewer side effects and higher tumor selectivity than those of traditional treatments [94]. Under the intense effect of light irradiation, the local activation of photosensitizers (PS) could trigger photochemical pathways and generate reactive oxygen species (ROS), leading to the induction of cell damage and final cell death [95]. Herein, PARPi was utilized to improve PDT efficacy via the PARP damage-repair signaling pathway. Nanocarriers are a great choice to simultaneously deliver PS and PARPi, and have been employed to enhance therapeutic activity.

Veliparib (PARPi) and methylene blue (MB, PS) were co-encapsulated in PLGA NPs to obtain a stable drug delivery system (termed VMB-NPs). VMB-NPs had a mean size of 90 nm with a polydispersity index of 0.08 ± 0.03, and a zeta potential of −3.7 ± 0.2 mV. VMB-NPs displayed controlled release profiles of MB and veliparib with an initial burst release and a delayed release over 450 h, which was a positive feature in the combination of VMB-NPs treatment and PDT. The initially released MB resulted in photodamages to cells, while veliparib released simultaneously and afterward inhibited the recovery of photodamaged cells, thus enhancing the efficiency of combined therapy. The VMB-NPs were demonstrated to enhance photoactivity since there was no cytotoxicity to B16F10-Nex2 cells observed in the dark, but cell viability obviously decreased after irradiation and treatment with VMB-NPs [96]. Hence, the strategy of combining PARP inhibition with PS photodamage in a nanosystem opened a new door to improving PDT efficacy for cancer treatment.

### 2.3. Others

There was an exemplary study about a hybrid nanosystem composed of PARPi combined with magnetic NPs for cancer treatment. The PARPi quercetin (Q) was conjugated with dextran-aldehyde (DA) to obtain amphiphilic QDA to assemble NPs. Furthermore, Cu(II) was encapsulated into QDA to enhance DNA cleavage, and the final CuQDA/IO@HA NPs were prepared after the dual modification of HA and superparamagnetic IO. With the CD44 receptor target of HA and the magnetic navigation of IO, the tumor selectivity and targeting ability were significantly improved. The results showed that CuQDA/IO@HA specifically targeted BRCA-mutant cancer cells and induced specific cytotoxicity in vitro, while it exhibited biocompatibility with normal cells. For BRCA-mutant xenograft mice that were treated with CuQDA/IO@HA and subjected to magnetic navigation, powerful PARP inhibition and DNA damage were clearly observed, and the median survival was significantly prolonged compared to that of plain Q treatment [97]. Hence, integrating inhibitors and metal ions into a nanomedicine it is a promising strategy to enhance the target capacity and efficiency of drugs.

In another project, Gonzales et al. entrapped a fluorescent PARP1 imaging agent into a nanoemulsion to obtain PARPi-FL NPs, a fluorescently labeled sensor for olaparib. This nanoemulsion protected the fluorescent PARPi from rapid washout in blood circulation and accumulated in solid tumors of small cell lung cancer, which effectively enhanced the circulation time and reduced off-target toxicity. The PARPi-FL in the tumor was able to localize to accordant targets in cell nuclei, where they bind to PARP1 and are released to play the key role of an imaging agent [98].

## 3. Conclusions and Prospects

The powerful antitumor efficacy of PARPi has been confirmed in many solid tumors. With the emergence of extra toxicity to normal tissues and drug resistance of tumor cells, NPs as a delivery platform successfully resolved these limitations. Designed PARPi delivery nanosystems that exhibit controlled release, size optimization, and target ligand modification are able to enhance selectivity to tumor cells, increase drug accumulation in tumor sites, and decrease off-target toxicities. Moreover, the combination of PARPi with chemotherapy, RT/PDT, and other therapies based on nanotechnology could further exhibit synergistic effects in cancer treatment. However, there are still some areas that need to be improved for the current PARPi nanosystems. For example, the designed NPs should have a more uniform particle size distribution, better long-term stability, and less degradable material in vivo. Moreover, to overcome PARPi resistance, the optimal combination of PARPi with other treatment agents need to be explored urgently. Altogether, PARPi-based nanosystems have shown an important and strong role in current cancer therapies, and are expected to have great potential in the future.

## Data Availability

Not applicable.
